# Perforation of the Colon by Small Foreign Bodies

**Published:** 1903-11-07

**Authors:** 


### PERFORATION OF THE COLON BY SMALL FOREIGN BODIES.

Many an instance has been reported of abdominal tumour in which, after opening the peritoneum, a condition has been found which to all appearances has been malignant disease of the bowel too advanced for removal, and yet in spite of the gloomy prognosis which has been given the patient has recovered. Hitherto no satisfactory explanation of these occurrences has been offered. Mr. Bland Sutton[1] considers that in some instances the condition has been caused by a small foreign body which has perforated the wall of the gut and become encysted, or which has been caught in a small pouch connected with the gut. He shows that in stout individuals after middle life there are occasionally found little pouch-like diverticula from the lumen of the large intestine projecting between the layers of the mesentery or into one of the thickened appendices epiploicæ, and he believes that small foreign bodies which have been accidentally swallowed may get caught in these sacculi and there set up chronic irritation. He gives two instances where this condition was actually found in patients upon whom he operated. In both cases small foreign bodies were discovered in the midst of an enlarged, thickened, and inflamed appendix epiploica, and in one of the cases the condition could not be distinguished at the time of the operation from cancer, and a resection of the affected portion of the bowel was performed. It will be interesting to note, now that attention has been drawn to the matter, whether or not it will fall to the lot of other surgeons to meet with and to recognise this peculiar pathological condition, which, in the light of Mr. Bland Sutton's observations, may prove to be one of considerable practical importance.

[1] Lancet, Oct. 24, 1903.

